# Generation and Evaluation of Recombinant Thermostable Newcastle Disease Virus Expressing the HA of H9N2 Avian Influenza Virus

**DOI:** 10.3390/v13081606

**Published:** 2021-08-13

**Authors:** Xiaorong Zhang, Zongyi Bo, Chenchen Meng, Yin Chen, Chengcheng Zhang, Yongzhong Cao, Yantao Wu

**Affiliations:** 1 Jiangsu Co-Innovation Center for the Prevention and Control of Animal Infectious Disease and Zoonoses, College of Veterinary Medicine, Yangzhou University, Yangzhou 225009, China; zxr@yzu.edu.cn (X.Z.); zybo@yzu.edu.cn (Z.B.); ccmeng971225@163.com (C.M.); chen123yin2021@163.com (Y.C.); zcc@yzu.edu.cn (C.Z.); 2 Joint International Research Laboratory of Agriculture and Agri-Product Safety, The Ministry of Education of China, Yangzhou University, Yangzhou 225009, China

**Keywords:** Newcastle disease virus, thermostability, H9N2 avian influenza virus, HA protein, transmembrane domain, recombinant virus

## Abstract

H9N2 avian influenza virus (AIV) has become endemic in many countries, causing great economic losses when co-infected with other pathogens. So far, several live vaccines based on Newcastle disease virus (NDV) vectors expressing influenza hemagglutinin (HA) have been developed. However, the thermostable recombinant NDV is rarely reported. In this study, using a thermostable NDV rAHR09 strain as the vector, three recombinant NDVs expressing native HA, chimeric HA ectodomain with transmembrane domain/C-terminal cytoplasmic tail domain from fusion protein of NDV, and HA ectodomain were generated, designated rAHR09-HA, rAHR09-HAF, and rAHR09-HAE. The MDT value of three recombinant NDVs was above 120 h, their ICPI value was about 0.03, and the recombinant NDVs were still infectious when treated for 100 min under 56 °C, which demonstrated that the recombinant NDVs kept the lentogenic and thermostable nature of rAHR09. The immunization data showed that rAHR09-HA and rAHR09-HAF induced a higher HI antibody titer against H9N2 AIV and NDV. After being challenged with H9N2 AIV, the rAHR09-HA and rAHR09-HAF could significantly reduce the virus shedding in cloacal and tracheal swab samples. Our results suggest that rAHR09-HA and rAHR09-HAF might be vaccine candidates against H9N2 AIV.

## 1. Introduction

H9N2 avian influenza virus (AIV) has low pathogenicity to chickens, only causing mild clinical symptoms. However, when co-infected with other pathogens, it can cause a synergistic pathogenic effect and lead to severe clinical symptoms and higher mortality [1,2]. To date, despite biosecurity measures, vaccination is still the main method used in the control of H9N2 AIV [3,4,5]. There are several kinds of virus vectors that have been used for the development of an AIV vaccine, such as fowlpox virus and Marek’s disease virus [6,7,8]. The virus vector-based vaccine can facilitate the immune procedure to reach the goal of “one immunization, multiple protection”. Indeed, there have been several reports that have showed that the NDV can be used to express the HA protein of AIV [9,10,11,12], and the replacement of the transmembrane (TM)/C-terminal cytoplasmic tail domain (CTD) of AIV HA protein with that of NDV F protein could improve the incorporation of the AIV HA protein into NDV virus particles [13].

NDV belongs to the genus Avulavirus, family Paramyxoviridae, with a nonsegmented, negative-sense, single-stranded RNA genome [14]. NDV is the causative agent of Newcastle disease (ND), which is a highly contagious and fatal viral disease. The thermostable NDV strain can facilitate its storage and transport, especially for the attenuated vaccine strain. Previously, one thermostable NDV was isolated in our lab, designated HR09, which could remain infectious for more than 1 hour under 56 °C treatment [15]. Through exchanging the proteins of thermostable HR09 and the La Sota strain, it was found that the residues 315 and 369 in the HN protein were responsible for the thermostability of HR09 [16]. The thermostable HR09 strain, which belongs to genotype VIII, is a mesogenic NDV strain. In order to use it as the vaccine vector, the prAHR09 plasmid that contained the HR09 full-length genome and the replaced cleavage site of the F gene from ^112^RRQKRF^117^ to ^112^GRQGRL^117^ were constructed in our lab. Finally, the attenuated NDV rAHR09 strain was obtained by co-transfecting the prAHR09 plasmid with the three helper plasmids pCI-NP, pCI-P, and pCI-L [15].

In this study, three thermostable recombinant NDVs that used the attenuated NDV rAHR09 strain as the expression vector to express different forms of the HA gene from H9N2 AIV were generated. Then, their thermostable characteristics, growth kinetics, virulence, and immunogenic of recombinant NDVs were evaluated. The results showed that all three recombinant NDVs showed a similar growth kinetics in CEF cells, had good thermostability, and induced a high level of NDV- and AIV-specific hemagglutination inhibition (HI) antibody titers. Among the three recombinant NDVs, the rAHR09-HA and rAHR09-HAF strains induced a higher antibody titer against H9N2 AIV and significantly reduced the level of H9N2 AIV virus shedding in both cloacal and tracheal swab samples.

## 2. Materials and Methods

### 2.1. Cells and Viruses

BSR-T7/5 cells, a selective cell line from BHK cells, which can express the T7 RNA polymerase, were generously provided by Dr. Zhigao Bu from the Harbin Veterinary Research Institute (HVRI), Chinese Academy of Agricultural Sciences (CAAS). Specific pathogen-free (SPF) embryonated chicken eggs were purchased from Boehringer Ingelheim Vital Biotechnology Co., Ltd. (Beijing, China). CEF was generated from 10-day-old embryonated chicken eggs. Cells were cultured with Dulbecco’s modified Eagle medium (Hyclone, Logan, UT, USA), supplemented with 10% fetal bovine serum (Hyclone) and 1% penicillin-streptomycin (ThermoFisher scientific, MA, USA) at 37 °C in a 5% CO_2_ incubator. The H9N2 AIV A/Chicken/Jiangsu/YZ1406/2014 strain (MZ424691) and the thermostable NDV HR09 strain (MF285077.1) were used in this study.

### 2.2. Antibodies and Reagents

The chicken anti-H9N2 AIV HA protein polyclonal antibody was generated in our lab. Rabbit anti-actin, horseradish peroxidase (HRP)-conjugated goat anti-chicken IgY, and HRP-conjugated goat anti-rabbit IgG were supplied by Merck (Darmstadt, Germany).

### 2.3. Plasmid Construction

The plasmid including Pme I restriction enzyme cutting site, prAHR09-Pme I, was constructed using overlapping PCR. The GFP or HA genes fused with gene-end (GE) and gene-start (GS) sequences of NDV, and Kozak sequences were amplified using overlapping PCR and ligated with the Pme I digested prAHR09-Pme I using the Seamless Cloning Kit (Vazyme, Nanjing, China). Finally, the plasmids contained the full-length NDV genome, and exogenous genes were constructed, designated prAHR09-HA, prAHR09-HAF, and prAHR09-HAE. All primer sequences used in the study are available upon request.

### 2.4. Rescue of Recombinant Virus

The recombinant NDVs were rescued as described previously [17]. Taking rAHR09-GFP as example, the main procedures are as shown below: firstly, the full-length NDV plasmid which contained the GFP expression cassette, designated prAHR09-GFP, was co-transfected with pCI-NP, pCI-P, and pCI-L using a Lipofectamine 3000 reagent (Thermo Fisher Scientific) in accordance with the manufacturer’s instructions, and 10% allantoic fluid from the SPF embryonated chicken eggs was separately added directly to the culture medium at 12 h and 36 h post-transfection. Whole-cell lysates were collected at 72 h post-transfection. After three freeze/thaw cycles, cell lysates were inoculated into 10-day-old SPF embryonated chicken eggs for 96 h, allantoic fluids were collected, and the titers of NDV were identified by HA assay.

### 2.5. RT-PCR and QRT-PCR

CEF cells were infected with different passages of recombinant NDVs for 72 h, and total RNAs were extracted using TRIzol (Merck, Darmstadt, Germany) and then reverse-transcribed to cDNA using the PrimeScript™ 1st Strand cDNA Synthesis Kit (Takara, Beijing, China). The full-length HA gene was amplified using the sense primer 5′-ATG GAG ACA GTA TCA CTA ATA ACT ATA C-3′ and the antisense primer 5′- GGG GGT TAT ATA CAA ATG TTG CAT CTG C-3′. The transmembrane replacement and deletion of HA genes were amplified using the same sense primer as the full-length HA gene, and with the antisense primer 5′-GGT CAT GTT CTT GTG GTG GCT C-3′ and 5′- GGG ATT TTG TAA GTT CCT TCA GAT TC-3′. The levels of virus shedding of H9N2 AIV were quantified via quantitative real-time RT-PCR using a Life Technology instrument. Briefly, cloacal swab and tracheal swab samples were collected at 3, 6, and 9 days post-challenge (dpc), then the RNA was extracted and reverse-transcribed to cDNA. The mRNA levels of AIV were determined using real-time RT-PCR assay as previously described [18].

### 2.6. Western Blot Analysis

The expression of the HA protein was detected using Western blot analysis. Briefly, CEF cells infected with different passages of recombinant NDVs were prepared in a 2×SDS lysis buffer and subjected to SDS–PAGE, then transferred to a polyvinylidene difluoride (PVDF) membrane (Pall Corporation, NY, USA) under 200 mA for 1.5 h. The membranes were blocked with 3% bovine serum albumin (BSA) in phosphate-buffered saline with 0.5% Tween-20 (PBST) for 4 h at 4 °C and then incubated with the primary antibody overnight at 4 °C; after washing with PBST 3 times, the membranes were incubated with a secondary antibody for 6 h at 4 °C. The specific bands were captured using an enhanced ECL reagent (Thermo Fisher Scientific, Waltham, USA).

### 2.7. Viral Growth Curve

The growth kinetics of different recombinant NDVs were measured using the TCID_50_ method. Briefly, monolayer CEF cells infected with different NDVs (0.1 MOI) were harvested at 24, 48, 72, and 96 h post-infection (hpi). The TCID_50_ of each sample was determined in CEF cells, and the results were calculated using the Reed and Muench method as described previously [19].

### 2.8. Thermostability Test

Each virus with 10^8^ HA titer at 1.0 mL per vial was submerged into a water bath and incubated at 56 °C for 30, 40, 50, 60, 80, 100, and 120 min. Then, the virus was transferred to an ice-cold water bath to stop heat treatment. The virus titer of each vial was measured using the HA method.

### 2.9. Virulence Determination

The virulence of each virus was assessed by applying the mean death time (MDT) test in 10-day-old SPF embryonated chicken eggs as described previously [20]. Briefly, the collected allantoic fluid from each recombinant NDV strain infected embryonated chicken eggs was diluted from 10^−5^ to 10^−12^ in 10-fold series, then 10-day-old SPF embryonated chicken eggs, 5 per group, were incubated with 100 μL of each dilution. Then, the challenged embryonated chicken eggs were incubated at 37 °C and candled every 12 h for 7 days, the death of each embryonated chicken eggs was recorded, and the MDT values were calculated. In addition, the intracerebral pathogenicity index (ICPI) of each virus was measured as described previously [21,22].

### 2.10. Chicken Immunization and Challenge

Eighty 7-day-old SPF chickens were randomly divided into four groups, and the SPF chickens per group were inoculated with 10^6^ EID_50_ of each recombinant NDV strain in 100 μL DMEM via the oculonasal route. In the control group, chickens were inoculated with 100 μL DMEM. The serum of chickens was harvested 7, 14, and 21 days post-immunization (dpi). At 21 dpi, the chickens in each group were challenged with the 10^8^EID_50_ H9N2 AIV YZ1406 strain. Clinical signs of the immunized and challenged chickens were monitored every day. Cloacal and tracheal swab samples were collected 3, 6, and 9 days post-challenge (dpc).

### 2.11. Virus Titer and Serum Antibody Titer Determination

The titer of recombinant NDVs was measured using a hemagglutination (HA) experiment, and the serum antibody titers against H9N2 AIV and NDV were determined via the hemagglutination inhibition (HI) method as described previously [23,24]. In addition, the virus titer was also measured using EID_50_ methods in 10-day-old embryonated chicken eggs as described preciously [19].

### 2.12. Ethics Statement

The operation and treatment of the animals were approved by the Institutional Animal Care and Use Committee of Yangzhou University (No. YZUDWLL-201802-012; Date: 12 February 2018).

### 2.13. Statistical Analysis

All experiments were performed at least three times. Data are shown as means ± standard deviations. The statistical significance between different groups was analyzed via Student’s *t*-test method in GraphPad Prism 7.0 (La Jolla, CA, USA).

## 3. Results

### 3.1. NDV rAHR09-GFP Recombinant Virus Constructed as the Basal Virus Vector

**Figure 1:**
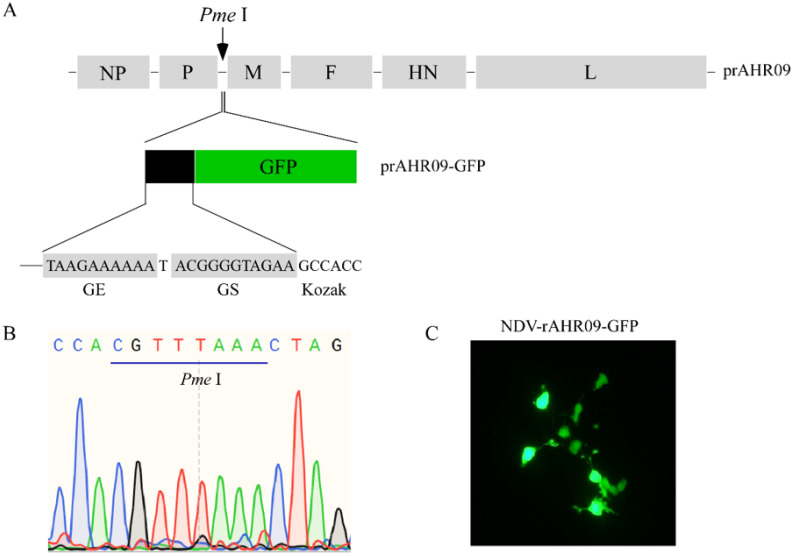
Construction of the attenuated NDV rAHR09-GFP recombinant virus. (**A**) Schematics for the construction of the NDV rAHR09-GFP recombinant virus. (**B**) Sequence identification of the insertion of *Pme* I enzyme cutting site. (**C**) BSR T7/5 cells in 6-well plates with 60% confluence were co-transfected with the prAHR09-GFP plasmid (0.8 μg) with pCI-NP (0.4 μg), pCI-P (0.2 μg), and pCI-L (0.2 μg) using Lipofectamine 3000 in accordance with the manufacturer’s instructions for 72 h; 12 and 36 h after transfection, 10% allantoic fluid was added to the cell culture. The GFP fluorescent image was acquired at 72 h post-transfection with a Nikon microscope (100×).

In order to construct a basal NDV vector to express the exogenous gene, we inserted a *Pme* I restriction enzyme cutting site between P and M genes on the prAHR09 plasmid. After prAHR09-*Pme* I was cut with *Pme* I, a seamless cloning method was used to insert the GFP expression cassette, which contained the GE, GS, and Kozak sequence ahead of the GFP gene. The schematics of the construction of prAHR09-GFP are shown in Figure 1A, and the verification of *Pme* I insertion into prAHR09 is shown in Figure 1B. Finally, the prAHR09-GFP plasmid was co-transfected with the three helper plasmids, and the fluorescence of the recombinant NDV rAHR09-GFP strain is shown in Figure 1C.

### 3.2. Construction and Verification of Recombinant NDVs by Replacing the GFP Gene with the H9N2 AIV HA Gene

**Figure 2:**
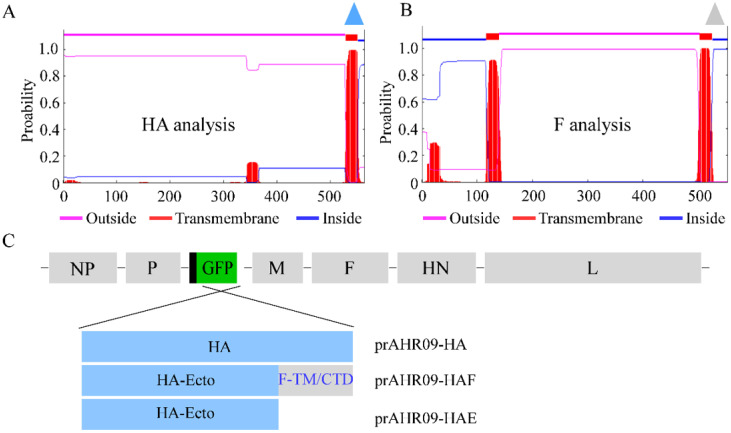
Schematics for the construction of three recombinant NDVs. (**A**,**B**) The transmembrane domain analysis of the H9N2 AIV HA protein and NDV HR09 F protein (http://www.cbs.dtu.dk/services/TMHMM/, accessed on 24 June 2017). (**C**) The schematics for the construction of the different forms of HA that replace the GFP gene in prAHR09-GFP plasmid. The plasmid containing the full-length HA was designated prAHR09-HA. The plasmid with the chimeric HA ectodomain fused with the transmembrane domain and C-terminal cytoplasmic tail domain from F protein of NDV was designated prAHR09-HAF. The plasmid with HA ectodomain was designated prAHR09-HAE.

To construct the recombinant NDVs that express the HA of H9N2 AIV, the transmembrane domain of the H9N2 AIV HA protein and NDV F protein was analyzed (Figure 2A,B). Then, three HA protein forms were designed to replace the GFP gene in the prAHR09-GFP plasmid (Figure 2C). The first one is the full-length HA gene, designated prAHR09-HA. The second one is the chimeric HA ectodomain (1–524 aa) fused with the C-terminal transmembrane domain (503–525 aa) and cytoplasmic tail domain (526–552 aa) from the F protein of NDV, designated prAHR09-HAF. The last one is HA ectodomain, designated prAHR09-HAE. After the plasmids were constructed, each of them was transfected with the three helper plasmids in BSR-T7/5 cells for 72 h. Then, the cell lysates were harvested and incubated in 10-day-old SPF embryonated chicken eggs for 4 days. The existence of recombinant NDVs in harvested allantoic fluids was checked using the HA test, and the results showed that all three recombinant NDVs were HA positive, which indicated that the three recombinant NDVs were rescued successfully.

**Figure 3:**
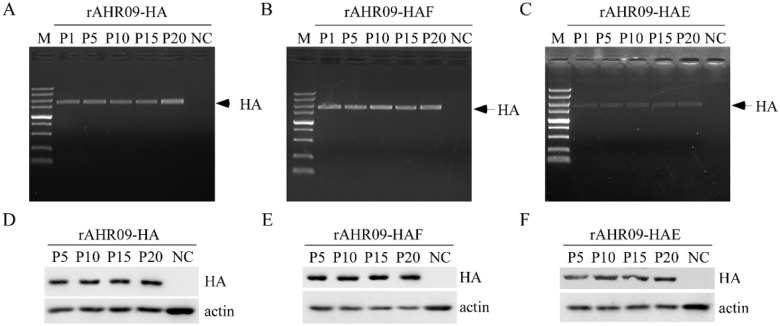
The recombinant NDVs were stable upon 20 passages in embryonated chicken eggs. The three plasmids prAHR09-HA, prAHR09-HAF, and prAHR09-HAE were transfected with the helper plasmids as shown in Figure 1C. The recombinant NDVs were obtained and designated rAHR09-HA, rAHR09-HAF, and rAHR09-HAE. Then, the recombinant NDVs were propagated in embryonated chicken eggs for 20 passages. (**A**–**C**) The RNA of passages 1, 5, 10, 15, and 20 was extracted and reverse-transcribed into cDNA, then PCR was performed to check for the existence of the HA gene. (**D**–**F**) The recombinant NDVs were propagated in CEF cells, and the whole-cell lysates of passages 5, 10, 15, and 20 were harvested and subjected to Western blot to check the expression of the HA protein.

To assess the stability of the recombinant NDVs, the recombinant NDVs were propagated in embryonated chicken eggs for 20 passages. Firstly, passages 1, 5, 10, 15, and 20 were chosen to check the existence of the HA gene using the PCR method. The results showed that rAHR09-HA (Figure 3A), rAHR09-HAF (Figure 3B), and rAHR09-HAE (Figure 3C) contained the HA gene in all passages. In addition, passages 5, 10, 15, and 20 were chosen and propagated in CEF cells to check the expression of the HA gene using the WB method, and the results showed that all the passages of the three recombinant rAHR09-HA (Figure 3D), rAHR09-HAF (Figure 3E), and rAHR09-HAE (Figure 3F) could express the HA protein, which indicated that the three recombinant NDVs could stably express the HA gene.

### 3.3. The Recombinant NDVs Kept a Similar Growth Kinetic and Thermostability with Their Parental Strain rAHR09

**Figure 4:**
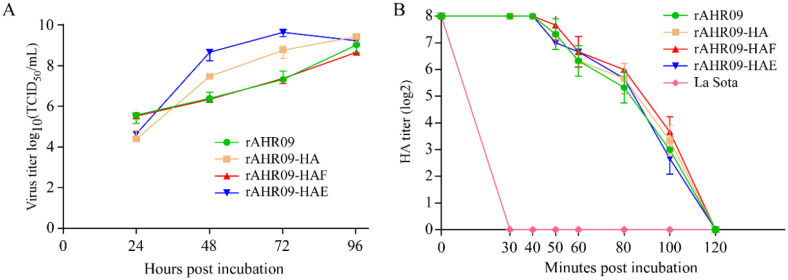
Biological characteristics identification of recombinant NDVs. (**A**) The CEF cells in 6-well plates were incubated with rAHR09, rAHR09-HA, rAHR09-HAF, and rAHR09-HAE at an MOI of 0.1, respectively. The cells’ supernatants were harvested at 24, 48, 72, and 96 hpi, then the virus titer was determined using the TCID_50_ method in CEF cells. (**B**) Each NDV recombinant virus was separately incubated for 30, 40, 50, 60, 80, 100, and 120 min under 56 °C. Then, the HA titer of each treated recombinant NDVs was measured.

To check whether the insertion of the HA gene influenced the biological characteristics of its parental strain NDV rAHR09, we measured their growth curves. The results showed that compared to rAHR09-HA and rAHR09-HAE, the rAHR09-HAF strain had similar growth kinetics with the parental strain rAHR09 in terms of replication cycles. At 48 and 72 hpi, the rAHR09-HA and rAHR09-HAE strains had a higher virus titer than the other two strains. What is more, all three recombinant NDVs and their parental strain had a similar virus titer at 96 hpi (Figure 4A).

As the rAHR09 strain was a thermostable NDV strain, we also checked whether the insertion of the HA gene could affect its thermostability. Each recombinant NDV with the 8log2 HA titer was treated for 30, 40, 50, 60, 80, 100, and 120 min under 56 °C. The HA experiment data showed that the HA titer of heat-treated recombinant NDVs showed no change when treated for 40 min, and recombinant NDVs could remain infectious as long as 100 min. As a negative control, the thermolabile NDV La Sota strain was not infectious when it was just treated for 30 min under 56 °C (Figure 4B). These data indicate that the recombinant NDVs still maintained good thermostability.

In addition, the virulence of the parental strain rAHR09 and the recombinant NDVs was evaluated by MDT and ICPI tests, and the results showed that the MDT value of all recombinant NDVs was longer than 120 h. The ICPI values of rAHR09, rAHR09-HA, rAHR09-HAF, and rAHR09-HAE were 0.032, 0.032, 0.029, and 0.027, respectively. These data indicate that all three recombinant NDVs belonged to lentogenic strains, and the insertion of H9N2 AIV HA gene did not increase their virulence compared to that of their parental rAHR09 strain.

### 3.4. Immunization with Recombinant NDVs Induced a High HI Antibody Titer against H9N2 AIV and NDV

**Figure 5:**
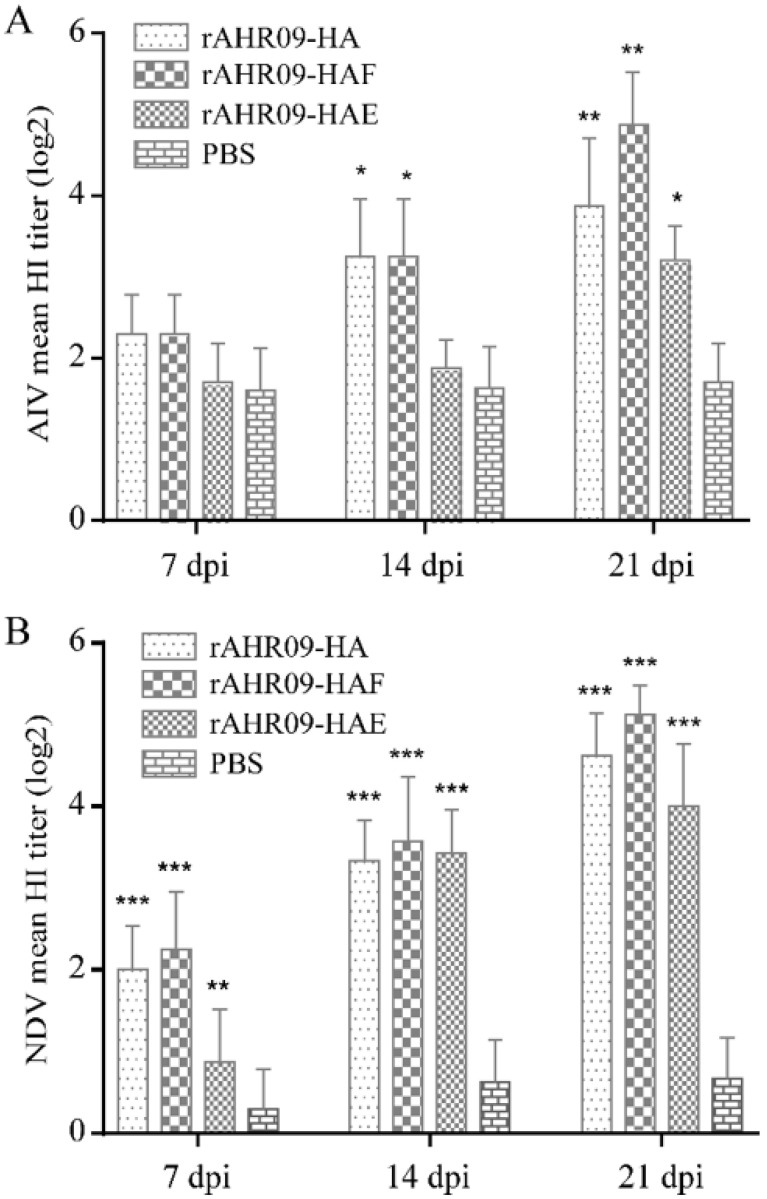
Serum antibody titer induced by recombinant NDVs in SPF chickens. Seven-day-old SPF chickens were separately immunized with 10^6^EID_50_ rAHR09-HA, rAHR09-HAF, and rAHR09-HAE. The serum was collected at 7, 14, and 21 dpi. (**A**) HI antibody titer against H9N2 AIV. (**B**) HI antibody titer against NDV. Values are shown as mean values ± standard deviation (mean ± SD) in each group (* *p* < 0.05, ** *p* < 0.01, *** *p* < 0.001).

Serum antibody titer is one of the most important criteria for evaluating the protective efficiency of the recombinant NDVs. In this study, to check whether recombinant NDVs could induce antibodies against H9N2 AIV and NDV, 7-day-old SPF chickens were separately immunized with 10^6^EID_50_ rAHR09-HA, rAHR09-HAF, and rAHR09-HAE through the oculonasal route. The serum was collected at 7, 14, and 21 dpi, and the serum antibody titer against AIV and NDV was measured using the HI method. The data showed that all of the three recombinant NDVs could induce the H9N2 AIV-specific antibody, and the antibody titer was increased from 7 to 21 dpi (Figure 5A). The same trend was observed for the antibody titer against NDV (Figure 5B). Among the three recombinant NDVs, the rAHR09-HA and rAHR09-HAF strains induced a higher HI titer against both NDV and H9N2 AIV.

Additionally, all three groups immunized with the recombinant NDVs did not show any clinical signs of NDV, which further demonstrated that the recombinant NDVs belonged to the lentogenic strain. These data demonstrated that the three recombinant NDVs could efficiently induce a high level of HI antibody titers against both H9N2 AIV and NDV.

### 3.5. Reduction of Virus Shedding after the Immunized Chickens Were Challenged with H9N2 AIV

**Figure 6:**
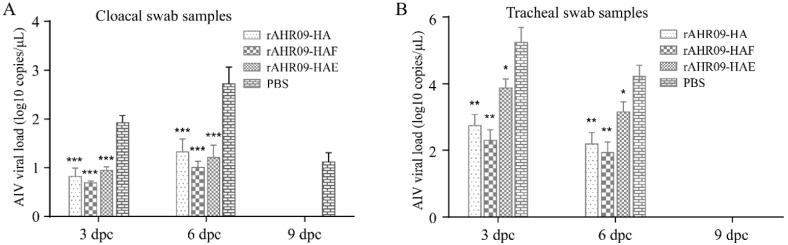
QRT-PCR quantitation of H9N2 AIV viral shedding in challenged chickens. The chickens were challenged with the 10^8^EID_50_ H9N2 AIV YZ1406 strain at 21 days post-immunization. Cloacal swab samples (**A**) and tracheal swab samples (**B**) were collected 3, 6, 9 days post-challenge, and the levels of H9N2 AIV viral RNA were quantitated via QRT-PCR. Values are shown as mean values ± standard deviation (mean ± SD) in each group (* *p* < 0.05, ** *p* < 0.01, *** *p* < 0.001).

H9N2 AIV belongs to low pathogenic AIV, so it is hard to evaluate the protective efficiency of the recombinant NDVs against the H9N2 AIV from the clinical signs. Reduction of virus shedding in vaccinated chickens after the AIV challenge was a commonly used criterion for evaluating the efficiency of the H9N2 AIV vaccine. To check whether the recombinant NDVs could protect vaccinated chickens against the challenge of H9N2 AIV, chickens were challenged with the 10^8^EID_50_ H9N2 AIV YZ1406 strain at 21 dpi. Cloacal and tracheal swab samples were collected at 3, 6, and 9 dpc, and then, H9N2 AIV viral RNA was quantitated via QRT-PCR. The results showed that all chickens remained healthy without any clinical signs after they were challenged with H9N2 AIV. In the control group, the levels of virus shedding in the cloacal swab samples were increased from 3 to 6 dpc, and then decreased from 6 to 9 dpc. Compared with the control group, all the three recombinant NDVs could significantly reduce the shedding of H9N2 AIV in cloacal swab samples at 3, 6, and 9 dpc, and the rAHR09-HAF strain immunized group showed the highest reduction level but no statistical differences with the rAHR09-HA group (Figure 6A). In the control group, the levels of virus shedding in tracheal swab samples were decreased from 3 to 6 dpc, and at 9 dpc, the AIV viral RNA could not be detected any more. The same results were achieved in the tracheal swab samples, where the levels of virus shedding were decreased in recombinant NDV-immunized groups, especially in the rAHR09-HA and rAHR09-HAF groups (Figure 6B).

## 4. Discussion

Despite the fact that the mortality caused by H9N2 AIV was no more than 20%, which usually leads to slight respiratory and egg-drop symptoms, H9N2 AIV was found to result in a secondary infection or generate a synergistic pathogenicity effect when the chickens were co-infected H9N2 AIV with other pathogens [25,26,27]. For example, it was reported that co-infection of H9N2 AIV with *Salmonella enteritidis* could lead to synergistic pathogenicity [28]. In addition, co-infection with infectious bronchitis virus with H9N2 AIV in commercial broiler chickens could cause serious clinical signs and high mortality [29]. All these reports showed that H9N2 AIV is an important disease that needs to be controlled in the poultry industry.

With the help of a reverse genetic system, NDV has been well studied not only as regards its pathogenesis and biological characteristics, but also its usage as a virus vector to express exogenous genes of other pathogens. For example, using NDV as the vector to express the human immunodeficiency virus type 1 (HIV-1), the gp160 envelope glycoprotein induced strong mucosal and serum antibody responses in guinea pigs [30]. When expressed in the NDV vector, ASFV p72 protein induced a high antibody titer of p72 specific IgG, and recombinant NDV-p72 virus also elicited T-cell proliferation and the secretion of IFN-γ [31]. All these studies showed that NDV could activate both humoral immunity and cellular immunity. In addition, there are several studies which used NDV as a virus vector to express the HA gene of H9N2 AIV, and recombinant viruses showed good protection against the challenge of NDV and H9N2 AIV at the same time [32]. In these studies, the backbone virus vectors were usually thermolabile NDV strains, such as La Sota strain, NDV Clone 30, or Herts/33 strain [32,33,34]. However, the thermostable NDV-based recombinant AIV vaccine candidate has rarely been studied.

Compared to other kinds of virus vectors, such as Marek’s disease virus or adenovirus, NDV has a small genomic RNA, which just encodes six proteins (NP-P-M-F-HN-L), so there will be little influence on the expression and immunization of the exogenous protein [35,36,37]. In theory, foreign genes can be inserted into the sequences between any two genes of NDV. However, because of the polar gradient transcription of NDV genome RNA, foreign genes are ideally inserted closely to the 3′ end of the NDV genome in order to be expressed at a high level [38,39]. With more and more studies focusing on the study of NDV recombinant virus, the site between the P and M genes has been considered an optimal selection for the insertion of foreign genes [40]. In this study, a thermostable attenuated NDV rAHR09 strain was used as the backbone to generate the recombinant NDV, and the three forms of the HA gene were inserted into the sites between the P and M genes (Figure 2B). After the recombinant NDVs were rescued, we also checked their stability and growth curves. The results showed that after 20 passages in embryonated chicken eggs, all three recombinant NDVs could stably express the HA protein (Figure 3D–F). The growth kinetic analysis showed that insertion of the HA gene had little influence on the growth of NDV, especially the rAHR09-HAF strain (Figure 4A), which might be due to the replacement of the HA transmembrane domain/C-terminal cytoplasmic tail domain with that of the NDV HR09 strain F protein.

For clinical usage, attenuated live NDV vaccine can be immunized via drinking water or the direct spray method, so it is favorable to maintain the infectivity of the NDV vaccine as long as possible. To achieve this goal, a thermostable NDV strain is necessary. Previously, a thermostable NDV HR09 strain, which could stay infectious for more than 100 min under 56 °C, was attenuated using a reverse genetic system in our lab [15,16,41]. Compared with the widely used La Sota strain, the attenuated rAHR09 strain could efficiently reduce the NDV virus shedding and provide full protection against the challenge of NDV [15]. Using this attenuated rAHR09 strain as the basal virus vector, three recombinant NDVs were constructed and treated for 30, 40, 50, 60, 80, 100, and 120 min under 56 °C. The results showed that all three recombinant NDVs could remain infectious for more than 100 min. By contrast, the thermolabile NDV La Sota strain could stay infectious for no more than 30 min (Figure 4B). These results demonstrated that the three recombinant NDVs still maintained good thermostability, which will relieve the cold chain transport bottlenecks.

To check whether the insertion of the H9N2 AIV HA gene affected the pathogenicity of the recombinant NDVs, the MDT and ICPI values of recombinant NDVs were measured. The data showed that the MDT values of rAHR09, rAHR09-HA, rAHR09-HAF, and rAHR09-HAE were all longer than 120 h, and their ICPI values were 0.032, 0.032, 0.029, and 0.027, respectively. These data demonstrated that all three recombinant NDVs belonged to lentogenic NDV, and insertion of the HA gene did not affect the pathogenicity of recombinant NDVs. By contrast, the MDT and ICPI values of commercial vaccine NDV La Sota strain were 115 h and 0.1, which indicates that the three recombinant NDVs were safe enough to be used as a vaccine on chickens [42].

The immune response induced by the vaccine is an important factor to be considered in vaccine development. In this study, chickens were immunized with each of the three recombinant NDVs, and the antibody titer was measured using the HI method. The data showed that all three recombinant NDVs could induce strong immune responses against both NDV and H9N2 AIV. Among these three recombinant NDVs, the rAHR09-HAF strain induced the highest HI antibody titer against both NDV and H9N2 AIV (Figure 5A,B). We also challenged the immunized chickens with H9N2 AIV, and the results showed that all three recombinant NDVs could reduce the shedding of the AIV in cloacal and tracheal swab samples, and the rAHR09-HAF strain produced results which were a little better than those of the other two recombinant NDVs (Figure 6A,B). These data demonstrated that the three recombinant NDVs could provide good protection against the challenge of H9N2 AIV. At present, the inactivated H9N2 vaccine is widely used and provides good protection against H9N2 AIV. However, the inactivated H9N2 vaccine can only induce the humoral immunity response, while the NDV-based recombinant virus can induce not only a humoral immunity response but also a cellular immunity response and even mucosal immunity response, so the vaccine developed in our study is a good supplement for the H9N2 inactivated vaccine [43,44]. For further clinical usage of this NDV-based recombinant H9N2 vaccine, we think a suitable method would be to use it as the prime immunization and then combine it with an inactivated H9N2 vaccine as the booster immunization, which will induce the humoral immunity response, cellular immunity response, and mucosal immunity response at the same time. We will perform studies to explore the further clinical use of this NDV-based H9N2 vaccine.

In summary, this study used a thermostable NDV rAHR09 strain as a vaccine vector to express three forms of the HA gene of H9N2 AIV. All three recombinant NDVs showed good stability in expressing the HA gene and maintained good thermostability. Among these three recombinant NDVs, the rAHR09-HA and rAHR09-HAF strains induced a higher HI antibody titer against NDV and H9N2 AIV, and they reduced the shedding of H9N2 AIV more efficiently compared to rAHR09-HAE. To summarize, the recombinant rAHR09-HA and rAHR09-HAF strains might be promising vaccine candidates for chickens against H9N2 AIV.

## 5. Conclusions

Three recombinant NDVs that contained different forms of the HA protein of H9N2 AIV were constructed using a reverse genetic system based on the thermostable attenuated NDV rAHR09 strain. All three recombinant NDVs were stable in expressing the HA gene upon 20 passages in embryonated chicken eggs, and virulence assay showed that all three recombinant NDVs belonged to lentogenic NDV. The immunization assay showed that both the rAHR09-HA and rAHR09-HAF strains induced a higher HI titer against NDV and H9N2 AIV, and they could efficiently reduce the shedding of AIV in cloacal and tracheal swab samples among the three recombinant NDVs. Together, this study demonstrated that the recombinant rAHR09-HA and rAHR09-HAF strains might be potential thermostable vaccine candidates to protect chickens against H9N2 AIV.

## Data Availability

Not applicable.
